# Harnessing Extended Reality for Neurocognitive Training in Chronic Pain: State of the Art, Opportunities, and Future Directions

**DOI:** 10.3390/healthcare13111338

**Published:** 2025-06-04

**Authors:** Javier Guerra-Armas, Alberto Roldán-Ruiz, Mar Flores-Cortes, Daniel S. Harvie

**Affiliations:** 1 Experimental Health Psychology, Maastricht University, 6200 Maastricht, The Netherlands; javier.guerra@uma.es; 2 Faculty of Health Sciences, Universidad de Malaga, 29071 Malaga, Spain; marflco@hotmail.com; 3 Clinimetry and Technological Development in Therapeutic Exercise Research Group (CLIDET), Universidad de Valencia, 46010 Valencia, Spain; 4 Departamento de Fisioterapia, Facultad de Ciencias de la Salud, Universidad Francisco de Vitoria, Pozuelo de Alarcon, 28223 Madrid, Spain; 5 Innovation, Implementation and Clinical Translation in Health (IIMPACT in Health), Allied Health and Human Performance, University of South Australia, Adelaide, SA 5001, Australia; daniel.harvie@unisa.edu.au

**Keywords:** chronic pain, neurocognitive impairments, extended reality, sensorimotor control, pain management, neurocognitive training, virtual reality

## Abstract

Chronic pain is a significant burden affecting more than 30% of people worldwide. Within the multiple biopsychosocial factors affected in people suffering from chronic pain, neurocognitive impairments represent a significant but often under-recognized aspect of the chronic pain experience that impacts daily life and healthcare. Multiple neurocognitive domains, including attention, executive function, learning, and memory, have been commonly associated with chronic pain. Within novel approaches, extended reality (XR) has been highlighted for its potential in chronic pain management. XR offers unique features to enhance traditional neurocognitive interventions, including dual tasks, gamification, ecological validity, and enriched experience, to increase engagement and motivation in rehabilitation. This systematic–narrative hybrid literature review aims to shed light on the potential benefits, challenges, and future directions of XR technology to address neurocognitive impairments associated with chronic pain. While preliminary evidence suggests that XR-based neurocognitive training may be beneficial in overcoming neurocognitive impairments found in chronic pain, some challenges still need to be addressed for effective translation into clinical practice. Within a transdiagnostic approach, XR-based neurocognitive training appears to be valuable across different diagnoses in chronic pain, wherein XR may emerge as a promising first-line intervention toward personalized multimodal management for chronic pain. Despite the rapid development of substantial growing evidence for XR, enhanced methodological rigor and reporting quality are recommended in future studies. More research is needed to fully understand the mechanisms and optimal application of XR-based neurocognitive training in different chronic pain conditions.

## 1. Introduction

**Figure 1:**
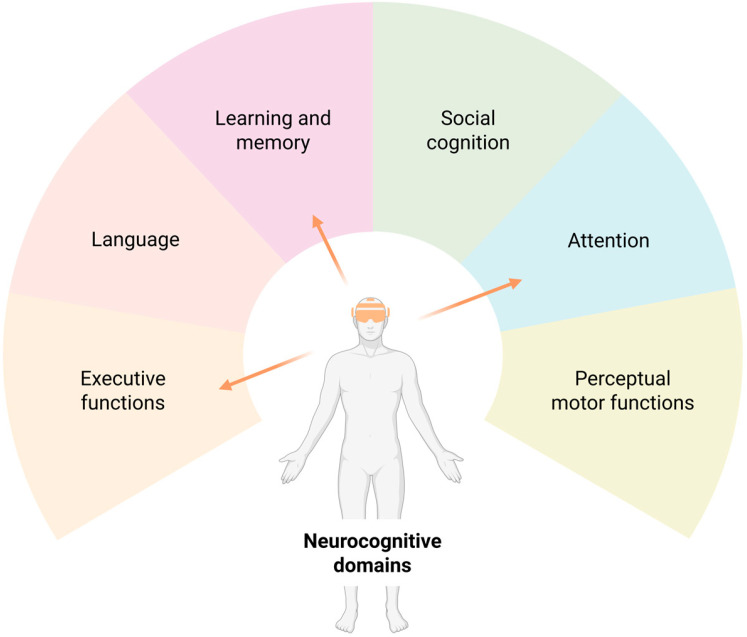
DSM-5 neurocognitive domains: Six key domains of cognitive function have been recognized in DSM-5, each of which has subdomains. Subdomains of neurocognitive functions include the following: (1) Executive function: Planning, Decision-making, Working memory, Responding to feedback, Inhibition, and Flexibility. (2) Language: Object naming, Word finding, Fluency, Grammar and syntax, and Receptive language. (3) Learning and memory: Free recall, Cued recall, Recognition memory, Semantic and autobiographical long-term memory, Implicit learning, and Language. (4) Social cognition: Recognition of emotions, Theory of mind, and Insight. (5) Attention: Sustained attention, Divided attention, Selective attention, and Processing speed. (6) Perceptual–motor function: Visual perception, Visuo-constructional reasoning, and Perceptual–motor coordination. Thus, pinpointing the domains and subdomains affected in a particular patients with chronic pain could help identify patients who may require additional intervention with XR to target neurocognitive impairment.

Pain is a complex experience involving deeply interconnected biopsychosocial dimensions [1]. According to the latest version of the International Classification of Diseases (ICD-11), chronic pain is defined as pain that persists and perseverates over a period of more than three months [2]. Thus, chronic pain is now considered a distinct condition that exerts an enormous individual and social burden, affecting more than 30% of people worldwide [3,4]. As a leading cause of disability, chronic pain interferes with an individual’s daily functioning and quality of life [4]. Within the multiple biopsychosocial factors affected in people living with chronic pain, neurocognitive impairments represent a significant but often under-recognized aspect of the chronic pain experience that interferes with activities of daily living (ADLs) [5,6]. A 20% prevalence of neurocognitive impairment has been reported in individuals with chronic pain [7], higher than in the general population [8]. However, systematic reviews have pointed out that these findings should be taken with caution given the low quality of the studies and the heterogeneity in the outcome measures used [9]. Although “neurocognitive functions” is a complex theoretical concept with no clear consensus on its definition or use in the literature, in this context, it refers to the mental processes involved in interpreting, planning, and carrying out actions within a specific context [10]. According to the consensus of the Neurocognitive Working Group of the Diagnostic and Statistical Manual of Mental Disorders 5th edition (DSM-5), six main domains of neurocognitive function can be identified [11] (Figure 1).

**Figure 2:**
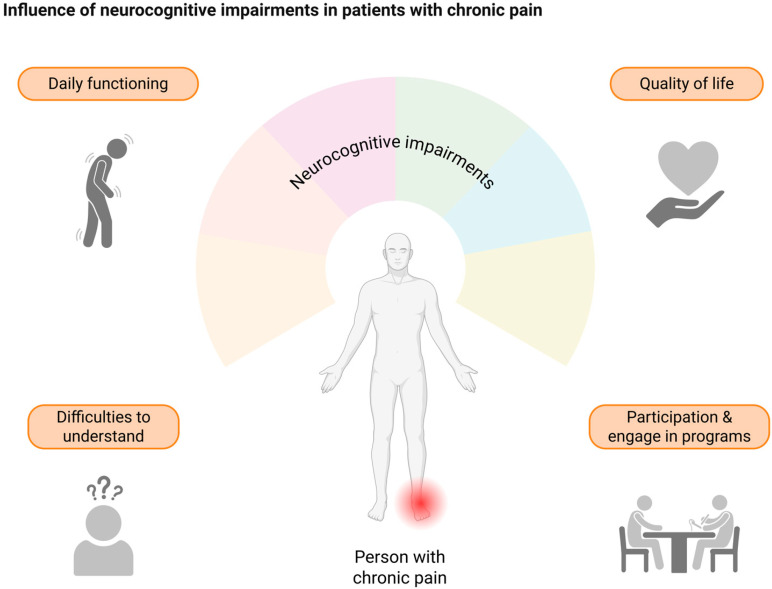
Interferences of neurocognitive impairments in activities of daily life and rehabilitation management. The impact of neurocognitive impairments on the quality of life of people suffering from chronic pain extends across multiple dimensions, affecting both activities of daily living and the receipt of healthcare.

The relationship between pain and neurocognitive function appears to be bidirectional, as chronic pain may disrupt cognitive processes, potentially exacerbating the pain experience [12,13]. It has been highlighted that neurocognitive functioning should be considered during the rehabilitation process of patients with chronic low back pain, as individuals with higher pain intensity had greater cognitive impairment than individuals with lower pain intensity [14]. However, these impairments show high variability and may be influenced by pain characteristics, psychological factors, and individual differences [15]. Likewise, comorbid affective disorders (such as depression and anxiety), sleep disturbance effects, and analgesic medication use, such as opioids, may further impact neurocognitive domains in people suffering from chronic pain [15]. While patinets with chronic pain demonstrate impairments across multiple neurocognitive functions, research has deepened into attention, learning and memory, perceptual–motor function, and executive function [16]. Such neurocognitive impairments associated with chronic pain have far-reaching consequences for daily functioning and quality of life [15] and may impact the ability to self-regulate pain and emotion [17] (Figure 2).

Clinical implications of neurocognitive impairments in chronic pain are significant and multidimensional, affecting various aspects of patient care, such as assessment and treatment outcome [18,19]. First, neurocognitive impairments may affect patients’ ability to accurately recall and report their pain experiences. Therefore, clinicians must take cognitive function into account when interpreting patient-reported outcomes [15]. In addition, patients with impaired neurocognitive functioning may have difficulties in understanding and complying within multimodal treatment programs, and it may also affect patients’ ability to participate and engage in physical therapy or pain education programs [5]. In this context, neurocognitive function may also play a role in the ability of patients with chronic pain to adhere to exercise programs. A recent study found that selective attention, response inhibition, and processing speed were strong predictors of engagement with an online pain self-management program [20]. In summary, impaired neurocognitive functioning appears to be common in patients with chronic pain, being associated with how much pain interferes with daily life, and represents a major obstacle for the rehabilitation of patients suffering from chronic pain.

### 1.1. Mechanism of Neurocognitive Impairments in Chronic Pain Populations

Research has already explored the common underlying mechanisms of pain and neurocognitive function to better understand both the nature and direction of this link [21,22,23,24]. Nevertheless, the relationship between chronic pain and neurocognitive impairment is multifactorial, involving multiple direct and indirect mechanisms. Based on recent research advances, different mechanisms have been hypothesized to understand neurocognitive impairment in adults with chronic pain: (1) attentional bias, (2) altered neural network activity and structural changes in the brain, (3) pain-related neurobiological processes, and (4) pain-related psychological factors [25]. Given the complexity of this topic, an integrated theory that acknowledges both of the following components—that pain drains cognitive resources and disrupts various brain areas and neural plasticity—has been proposed [16,23,26]. However, further research is needed to fully understand these interactions and develop targeted interventions that address both pain relief and neurocognitive functioning in patients with chronic pain.

### 1.2. Sensorimotor Influence of Neurocognitive Impairments in People with Pain

According to the “***Triple network model***” of De Ridder et al. (2022), the embodied pain experience may subsequently lead to physical and cognitive disability, likely being mediated through neuroplastic brain changes [27]. Whereas core domains of pain and physical function must be considered in chronic pain [28], a variety of changes in sensorimotor control in pain populations have been previously described [29]. Patients with chronic pain, who have fewer cognitive resources, may exhibit poorer sensorimotor goal-directed behavior and motor decision-making [15]. These changes impact how individuals with chronic pain interact with/and respond to their environment in daily life [14].

**Figure 3:**
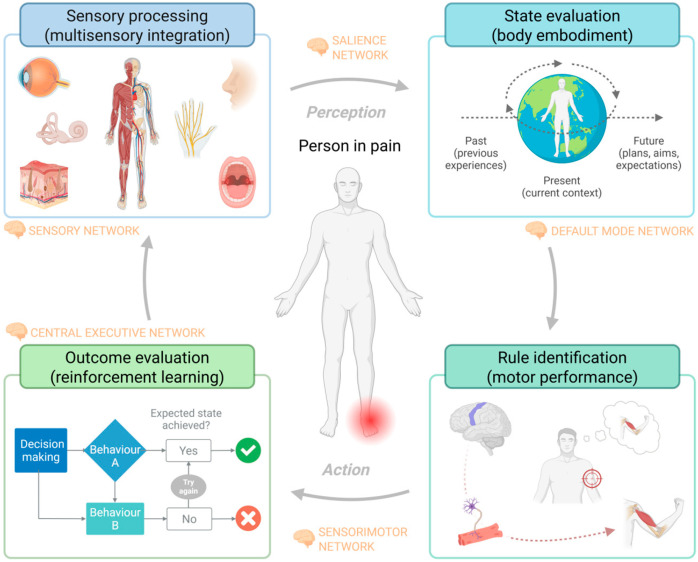
The sensory–motor close-loop perception model in chronic pain adapted from Guerra-Armas, J., Flores-Cortes, M., Pineda-Galan, C., Luque-Suarez, A., & La Touche, R. (2023). Role of immersive virtual reality in motor behavior decision-making in patients with chronic pain. *Brain Sciences*, 13(4), 617 [33]: Sensorimotor behaviors are “closed-loop” processes in which sensory feedback modulates behavioral output and in which regulatory and driving mechanisms via the cognitive system are involved in adapting movements to the demands and tasks of the environment. Consequently, the sensorimotor control system usually operates below our conscious level, and we become aware of the complex interaction between desired and actual movements only when there is an irregularity or incongruence in the system [34]. Thus, any deficit or impairment in sensory and/or neurocognitive processing may impact the functioning of people suffering from chronic pain.

Contemporary models of sensorimotor control in goal-directed behavior delineate distinct sensory-perceptual, neurocognitive, and motor processes [30]. Thus, the “***close-loop perception***” theory offers insight into how organisms acquire information from the environment, emphasizing that action plays a critical role in perception (Figure 3) [31]. Therefore, neurocognitive functions are crucial components of sensorimotor control in pain experiences, and these require cognitive evaluation, motor planification and prediction, learning, recall of past experiences, and active decision-making [32]. In summary, while further understanding of the interconnectedness between sensory, neurocognitive, and motor dimensions in people in pain is warranted, potential impairments in any of these dimensions should be considered in rehabilitation in chronic pain.

### 1.3. Chronic Pain Conditions Showing Neurocognitive Impairment

Chronic pain conditions are classified into chronic primary and chronic secondary pain [2]. There is growing acknowledgement that many, if not most, common chronic conditions are heterogeneous, often exhibiting significant overlap with other prevalent conditions, as well as being influenced by multiple biopsychosocial factors [35]. While diagnostic categories are useful for classifying and reporting clinical patterns, there is an increasing emphasis on the assessment of underlying diagnostic domains that share commonalities across multiple chronic pain conditions [36]. Such a transdiagnostic approach is particularly valuable in the study of overlapping symptoms as it can aid in the identification of shared mechanistic processes and better inform the development of novel therapeutic approaches [37]. Within this perspective, neurocognitive impairments have been reported in a number of conditions related to chronic pain such nociplastic pain [38], neuropathic pain [39], musculoskeletal pain [39], secondary headache or orofacial pain [40], cancer-related pain [41,42], post-surgical pain [43,44], sports injury-related pain [45], post-stroke pain [46], complex regional pain syndrome [47], opioid-related use disorders [48], and the elderly population [49].

### 1.4. Neurocognitive Training in Chronic Pain

The multidimensional and transdiagnostic nature of pain calls for multimodal and highly tailored care in chronic pain [4]. However, a recent systematic review found a lack of evidence on which interventions targeting neurocognitive functions are most appropriate in the chronic pain population [50]. Due to these concerns, emerging technologies have been identified as a promising area for tackling chronic pain [51]. Nonetheless, the burden of neurocognitive impairments in those who suffer from chronic pain and the use of extended reality (XR) and, more specifically, XR-based neurocognitive training remain seldom explored in chronic pain. Extended reality (XR) is an umbrella term for all immersive technologies, including current applications such as Augmented Reality (AR), virtual reality (VR), or Mixed Reality (MR) [52]. VR creates a three-dimensional virtual environment, where the user is embedded in losing “awareness” of the real world [53]. AR overlays digital elements within a real context, thereby enriching the user’s perception of reality [54]. MR combines the two, including aspects of the real world and the virtual world [55]. It has been suggested that the lack of uniform terminology can be attributed to several factors [56]: (1) the rapid development of technology means that new innovations are constantly emerging, often outpacing the adoption of up-to-date definitions; (2) research into XR-related taxonomy comes from a variety of disciplinary perspectives. XR-based neurocognitive interventions have been designed to overcome impaired neurocognitive functioning in rehabilitation and exhibited the potential to augment traditional neurocognitive training intervention [57]. However, this approach has been underused in chronic pain, where most studies in the pain field have focused on passive distraction [58], often not paired with neurocognitive tasks, despite the greater hypoalgesic effect [59,60,61,62]. Therefore, XR neurocognitive training may provide a novel therapeutic medium aimed at pain relief and tackling neurocognitive impairments related to chronic pain.

Given the knowledge gaps, a question arises: what does XR add to neurocognitive rehabilitation in populations with chronic pain conditions? This study therefore aims to provide an overview of XR-based neurocognitive training currently used to guide and evaluate scientific innovations in digital rehabilitation interventions for people with chronic pain. In addition, benefits, challenges, and future directions of XR-based neurocognitive training are presented, and recommendations for future digital health innovation are provided.

## 2. Materials and Methods

This review aims to shed light on gaps in the literature and synthesize the state of the science of XR-based neurocognitive training in people suffering from chronic pain. We provide a broad overview and narrative summary to highlight the potential benefits and challenges of XR technology to address neurocognitive impairments associated with the chronic pain experience. This manuscript follows a systematic–narrative hybrid literature review methodology to ensure the quality of our review. Six elements of the methodology proposed by Turnbull et al. (2023) [57] were followed: (1) research questions; (2) justification; (3) literature sources; (4) search parameters; (5) data cleaning; (6) information synthesis.

### 2.1. Research Question

The following questions were formulated to guide this review:

1. What is the available evidence on XR-based neurocognitive training in chronic pain?

2. Which are the benefits, challenges, and future directions underlying XR-based neurocognitive training in people suffering from chronic pain?

### 2.2. Literature Sources

We conducted an electronic search for articles published up to 12 April 2025 by searching the following electronic databases: PubMed, Google Scholar, and SCOPUS.

### 2.3. Search Parameters

The following search terms were used and combined using Boolean operators, focused on main components: ‘extended reality AND chronic pain’; ‘extended reality AND chronic pain AND neurocognitive training’; ‘virtual reality AND chronic pain AND neurocognitive training’, and ‘neurocognitive impairments AND chronic pain’. The resulting research included systematic reviews, narrative reviews, meta-analyses, and observational and empirical articles from inception to 30 March 2025.

### 2.4. Data Cleaning

Article selection criteria included the following: (a) in English, (b) peer-reviewed journal articles, (c) studies published up to 30 March 2025, (d) involving adults ≥ 18 years old, and (d) related to neurocognitive function or neurocognitive impairments in chronic pain and extended reality or virtual reality and neurocognitive training in chronic pain. The exclusion criteria were as follows: (a) studies that do not include neurocognitive outcome variables; (b) experimental or acute pain; and (c) preprints. After identifying studies fulfilling the inclusion criteria, titles, abstracts, and citation information obtained through the database search were exported to Mendeley Reference Manager system (Elsevier©, Netherlands), where duplicate records were manually identified and removed. First, potential articles were screened by title and abstract, then full texts of potentially eligible studies were obtained and evaluated for final synthesis by a single author (J.G.), and a second reviewer verified the selection to ensure the accuracy of the selection. Finally, studies that aimed to identify potential positive effects of extended reality targeting pain-related neurocognitive functions were included in the final synthesis (*n* = 23).

## 3. Summary of Findings

XR-based neurocognitive interventions have been designed to overcome impaired neurocognitive functioning in rehabilitation and exhibited the potential to augment traditional neurocognitive training intervention [58]. However, this approach has been underused in chronic pain, where most studies in the pain field have focused on passive distraction [59], often not paired with neurocognitive tasks, despite the greater hypoalgesic effect [60,61,62,63]. Therefore, XR neurocognitive training may provide a novel therapeutic medium aimed at pain relief and tackling neurocognitive impairments related to chronic pain.

A recent review presents an overview of benefits and barriers of the use of XR applied in rehabilitation, showing a growing interest in this technology within the field [64]. It has been suggested that technology-based approaches, such as XR, bring advantages over conventional rehabilitation, including enhanced individual motivation to exercise through attractive environments and positive rewards [65]. Accordingly, treatments delivered using XR are emerging as a promising non-pharmacological intervention in patients suffering from chronic pain with a medium-to-large effect size in pain intensity [66,67,68,69,70,71]. Likewise, XR has shown the potential to reduce the need for opioid medications by showing positive effects on disability, fear of movement, and negative affect in chronic pain populations [70,72,73,74,75,76]. While XR was largely unknown to patients with chronic pain and poorly implemented in routine clinical practice, a recent study reveals that patients consider it a promising strategy for low-risk remote therapy, valuing aspects such as portability, minimal side effects, and immersive experiences [77]. Besides pain effects, it has been highlighted that XR, including VR, has shown extensive benefits in the field of cognitive rehabilitation [78,79,80,81,82]. XR-targeted experiences may exert their therapeutic effects by triggering activity in brain regions involved in the descending pain modulation system, which is mediated by attention, emotion, and memory [59]. Likewise, the effects of neurocognitive interventions may be attributed to neuroplastic changes in nociceptive and non-nociceptive regions within the brain, which are correlated with reductions in pain intensity, pain-related cognition, and pain-related anxiety [83]. Recently, these potential mechanisms have been reported in a study by Ceko et al. (2022), who found that an immersive VR program in patients with chronic low back pain revealed somatosensory and prefrontal brain network changes that were associated with reduced pain intensity and pain interference, as well as improvements in disability, fear of movement, and pain-related worries [84]. Therefore, XR-based neurocognitive interventions targeting several specific neurocognitive functions might have a positive effect on chronic pain, as shown in Table 1.

### XR-Based Neurocognitive Training: Benefits and Challenges

Biological, neurophysiological, and psychological effects of physical activity (PA) and exercise on cognitive functioning have all been extensively studied in chronic disease populations [103,104,105]. Within the multiple possibilities covered by exercise, neurocognitive training refers to interventions using cognitive tasks or intellectually demanding activities, the goal of which is to enhance general cognitive ability [106,107] (e.g., attention, memory, executive function) by engaging users in cognitively demanding tasks and/or repetitive task-oriented exercises [108]. Both physical activity and neurocognitive training are common non-pharmacological interventional strategies that can mitigate the decline in cognitive and physical performance [109]. Neurocognitive training was recommended by the World Health Organization (WHO) for elderly people with normal cognition and mild cognitive impairment in order to reduce the risk of cognitive decline [110]. Depending on its goals, neurocognitive training follows different approaches, which can be broadly classified into process-based and strategy-based training paradigms [106]. Whereas the process-based approach involves the repeated performance (i.e., practice) of tasks that require executive function, strategy-based approaches use more explicit instructions for completing these tasks. However, due to the high degree of heterogeneity between studies in terms of sample size, functional measures used, intervention sessions (number, timing, and duration), and outcomes, the specific types of neurocognitive training programs that are most effective have not been established [111]. In recent years, the integration of digital health technology in neurocognitive training has emerged as a pioneering approach, revolutionizing cognitive treatments [112,113].

While neurocognitive training has received growing interest in the literature, the role of XR in the treatment of cognitive deficits has also gained significant attention in the scientific community [114]. Some authors have suggested that XR-based rehabilitation should be implemented within a multimodal rehabilitative approach and considered an effective treatment strategy for neurocognitive disorders [82]. Although the implementation of non-immersive VR in neurocognitive training, by using devices such as the Nintendo Wii or Microsoft Kinect, has been widely considered in the fields of neurorehabilitation and geriatrics, an increasing number of studies including immersive technologies have also been conducted to enhance outcomes in clinical populations [115,116]. It has been suggested that more immersive modalities of XR could offer further benefits alongside the effects already exhibited by non-immersive VR [117].

XR-based neurocognitive training enables rehabilitation programs including dual-task (motor and cognitive) training by interacting with virtual elements and/or introducing them into immersive environments to modulate the pain experience and to enhance neurocognitive functioning, brain plasticity, and ADLs [118]. Dual-task training has been defined as the concurrent performance of two tasks with distinct and separate goals. Meta-analytic evidence suggests that dual-task motor-cognitive training is the most effective type of training for improving neurocognitive function in healthy older adults [119]. Incorporating dual-task exercises that require neurocognitive processing has been suggested to be more advantageous than “classical” dual-task approaches or sequential motor-cognitive training in terms of enhancing brain neuroplasticity effects [120]. Liao et al. (2019) examined the effect of a dual-task VR exercise program incorporating both physical and cognitive training [121]. Their results showed that both VR and non-VR programs improved executive function and gait performance, comparable to combined physical and neurocognitive training, but dual-task performance was significantly improved only in the VR group. Despite these promising findings, it is still required to further identify what type and dosage of dual-task motor-cognitive training would potentially be most useful for enhancing neurocognitive impairments in the context of rehabilitation.

Another advantage offered by XR technology is gamification. It has been suggested that introducing gamification into rehabilitation may improve outcomes in health, well-being, and quality of life, as well as improving motivation for and adherence to exercise-based therapy [122]. While poor adherence to treatment has been reported to have negative effects on outcomes during rehabilitation interventions [123], XR-based interventions are underpinned by research because this technology is engaging and motivating [124]. Within neurocognitive functioning, the previous literature revealed that gaming experience results in faster reaction times and processing speeds, improved attention, improved decision-making, and improved multitasking abilities compared to non-gamified interventions [125,126,127]. These positive effects have even been proven in individuals with major neurocognitive disorders [128]. Thus, gamification offers “the unique opportunity for patients to interact in an enriched environment, providing structured, scalable training opportunities augmented by multi-sensory feedback to enhance pain relief and neuroplasticity through repeated practice” [129]. Although gamification is not specific to XR, harnessing motivation through XR-based neurocognitive training may improve outcomes and increase engagement and overall satisfaction [124].

A further benefit of XR-based neurocognitive training is increased ecological validity, task transfer, and increased control and standardization in rehabilitation interventions [130]. While screen-based applications such as non-immersive VR may improve performance in cognitive functioning, they are often criticized for their poor transferability to activities of daily living [108]. XR-based intervention could help to ecologically develop rehabilitation training by enabling a multimodal setting that is similar to situations that patients might encounter in their daily lives [78]. Such improved ecological validity may lead to specific emotional, cognitive, behavioral, and physiological responses [124]. Moreover, it has been identified that successful XR outcomes are largely influenced by the patient’s ability to use the XR systems independently, particularly in relation to the safety of use, subject experience, and ease of use of the devices [131]. Recent advances in XR technology enable more user-friendly interfaces and greater willingness to use, which reduce these barriers.

**Figure 4:**
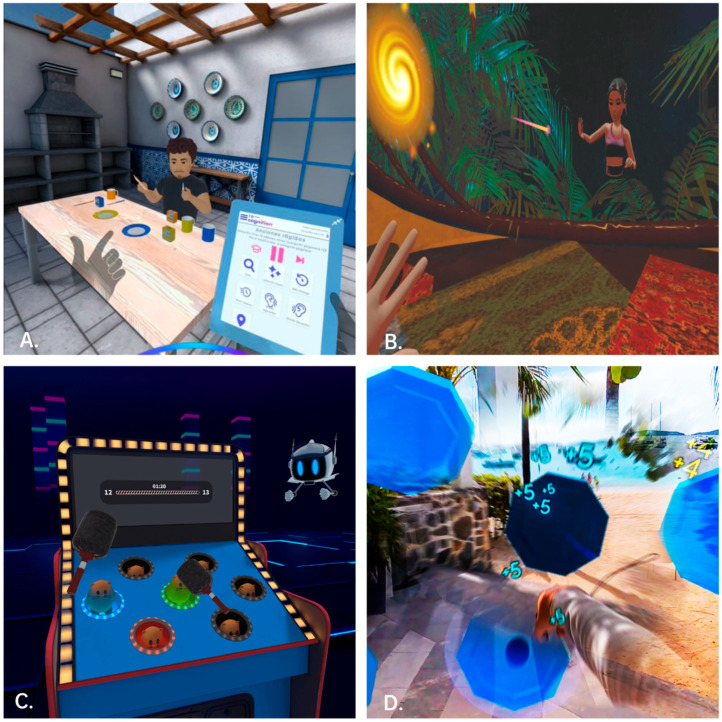
XR-based neurocognitive training available for chronic pain management: All games have multi-sensory inputs (vision and sound), high-quality graphics, and head and/or hand tracking, allowing for a highly interactive and gamified experience to enhance therapy engagement. (**A**) Recognition^®^ (Recognition, Granada, Spain); (**B**) Dynamics PainRehab^®^ (Dynamics VR Rehab, Seville, Spain); (**C**) Enhance^®^ (Virtualeap, Lisbon, Portugal); (**D**) Reflexion GO VR^®^ (Reflexion, Pennsylvania, United States). All images have been reproduced with the permission of the respective company, and all rights to these images are owned by the respective rights holders.

Whereas benefits have been outlined, multiple barriers have been identified for the successful implementation of XR in rehabilitation practice: equipment and costs, unfamiliarity with XR, adverse effects such as cybersickness, and the lack of standardized methods and protocols for XR clinical applications [64,132,133]. A major challenge in clinical implementation may be the limited availability of suitable games for rehabilitation, which is among the main barriers to the use of XR in rehabilitation [64,134,135]. While the number of available software is growing [56], few have been scientifically validated, as such software is expensive to build and maintain, and many companies lack sustainable sources of finance [135]. Funding mechanisms and collaboration with stakeholders are needed to support the long-term implementation of XR-based treatments [124]. Likewise, high costs are often identified as an obstacle for both providers and patients, which poses another challenge for scalability and clinical implementation [134]. Despite the increasing affordability and improving quality of XR systems, there is still uncertainty about the future direction toward broader clinical use. To overcome these challenges, in recent years, commercially available XR-based neurocognitive training has begun to offer short daily games designed to train and track neurocognitive domains such as memory, attention, goal-directed tasks, and perceptual–motor function [108]. Such applications of XR, in both VR and MR, may be easily implemented in clinical settings, due to the lower cost of the technology as well as the availability of software designed for neurocognitive training (Figure 4). A personalized multimodal approach for individuals with chronic pain including value-based goals has been encouraged [136]. Considering current technological advances, it can be assumed that XR is expected to expand into multimodal management programs but also operate as a stand-alone intervention for certain individuals. Nevertheless, further studies are still needed to examine the additional value of XR-based interventions in neurocognitive impairment compared to conventional interventions.

Today, chronic pain is considered a complex biopsychosocial phenomenon in which different factors interact with each other, influencing both the onset of symptoms and the prognostic course of the patient’s condition. Recently, an emerging “transdiagnostic” approach postulates that certain “vulnerability factors” contribute to the development and maintenance of various observed symptoms [137]. In the field of pain, adopting a transdiagnostic approach offers many clinical opportunities to identify modifiable vulnerability factors, which may provide a basis for targeted interventions to prevent the development of chronic pain and disability [138]. A paradigm shift from a tissue- and disease-based approach toward personalized multimodal intervention for chronic pain has been encouraged [136]. To this end, combining XR and artificial intelligence (AI) in neurocognitive training for pain relief has been recently suggested [139]. Although these technological innovations are still very much at a stage of infancy, the synergistic integration of AI with XR promises augmented XR-based neurocognitive training, thus tailoring therapeutic approaches to individual patient profiles, mechanisms of action, and the multiple biopsychosocial factors involved [139]. Therefore, harnessing potential benefits offered by XR-based neurocognitive training may offer a transdiagnostic tool in the management of chronic pain to address significant vulnerability factors such as neurocognitive impairments reported in multiple conditions.

## 4. Future Directions

The potential of XR-based neurocognitive training in chronic pain management has been highlighted in this review, but some of the challenges for effective translation into clinical practice still need to be addressed. More research is needed to fully understand the long-term neurocognitive impacts of XR-based interventions. Future studies should focus on the following:

- Larger sample sizes and longer follow-up times (i.e., 6–12 months) to improve the findings’ trustworthiness.
- Evaluations of the additional value of XR-based neurocognitive training in chronic pain compared to usual care.
- Because of the lack of and need for rigorous randomized controlled trials to examine the efficacy of XR compared to usual care or a wait-and-see approach, we encourage studies that would guide stakeholders in implementing XR-based neurocognitive training in daily practice. Future XR research would benefit from the development of standardized outcome measures and reporting frameworks. Initiatives such as the CONSORT extensions for non-pharmacologic interventions may serve as useful models.
- Standardized cognitive assessment tools to measure specific cognitive domains over time.
- Establishment of proper dosimetry and user experiences to guide evidence-based XR-based neurocognitive training in clinical practice.

Our results suggest that intervention delivered using XR can improve neurocognitive domains, including attention, memory, or executive function. Interestingly, an emerging insight derived from the transdiagnostic approach has been proposed in the present review: XR-based neurocognitive training appears to be valuable for neurocognitive training across different diagnoses in chronic pain. However, individual responses might differ according to different clinical profiles, and, as a result, more studies are recommended to fully understand individual differences when managing chronic pain. Further research needs to identify suitable candidates for XR-based treatments through the analysis of individual phenotypes and predictors of the response to intervention. A potential implication is developing more personalized training geared toward the specific neuropsychological profile and neurocognitive impairments that each patient presents in ADLs [140]. Moreover, the particular pain phenotype (nociceptive, neuropathic, and nociplastic) is relevant, as the mechanisms underlying each phenotype might greatly impact the magnitude of XR effects. The use of phenotyping to guide diagnosis, prognosis, and management has been recommended in chronic pain given the high heterogeneity of clinical pictures, high rate of recurrent symptoms, variability in pain trajectories, and uncertainty surrounding management [141,142,143]. Linton et al. (2024) [144] highlighted that identifying the main goals in people suffering from chronic pain is the first step to advance into precision medicine in rehabilitation [143,145]. Further studies are required to subcategorize individuals with chronic pain to better comprehend their response to XR-based neurocognitive training and thereby develop personalized strategies for each phenotype [51,146]. Nevertheless, it is noteworthy that currently, few studies directly compare the same type of intervention delivered with and without XR, which would help clarify the specific added value of XR-based neurocognitive training over its real-world counterparts. Similarly, there is a lack of studies comparing XR interventions with traditional computer-based cognitive training programs, limiting our understanding of their relative efficacy.

Finally, the available scientific evidence on XR in chronic pain management is heterogeneous, not only in terms of the hardware used (e.g., immersive or non-immersive technology) but also in terms of study design, methodological quality, sample size, clinical pain profiles, factors related to the user experience, and the dosimetry of XR intervention [20,36]. Further investigations are needed for the establishment of effective XR-based neurocognitive training components (i.e., task and training variables such as frequency, intensity, duration or volume of training, and type and content of specific XR scenarios) [86]. Transparent, accurate, and comprehensive reporting helps researchers replicate methods and designs, helps clinicians understand what was done and why, and helps stakeholders and policymakers implement results into clinical practice [147]. Thus, we recommend enhancing methodological and reporting quality following international guidelines such the TIDieR checklist for the description of trial interventions [148], CONSORT for randomized clinical trial reporting [149], and PRISMA and PERSiST for systematic review [147] to improve the comparability of studies, ultimately leading to more solid conclusions on XR’s effectiveness in chronic pain management.

## 5. Conclusions

While neurocognitive impairments are an important component within chronic pain, they have received little attention within multimodal pain management. According to this review, the use of XR in neurocognitive training in chronic pain is in its early stages. XR-based neurocognitive training offers a promising tool for the multimodal management of chronic pain. By harnessing some of the benefits provided by this emerging technology, XR-based neurocognitive training enhances patient engagement and motivation, potentially leading to reduced pain interference with activities of daily living. At present, commercially available XR-based neurocognitive training may reduce barriers for clinical implementation, while research may lead to personalized strategies for chronic pain phenotypes. While there is preliminary evidence suggesting the potential of XR-based neurocognitive training, more high-quality, comprehensive, and long-term follow-up studies are needed to clarify effectiveness.

## Figures and Tables

**Table 1:** Available evidence about the potential of extended reality targeting pain-related neurocognitive functions.

Pain Targets	Authors	Key Findings in XR
Attention	Hoffman et al., 2024 [85] Holzer et al., 2024 [86] Gamitto et al., 2020 [87] Hoffman et al.,2021 [88]	Redirects attention away from pain Improves focus on non-pain tasks Reduces pain intensity
Executive Function	Holzer et al., 2024 [86] Riva et al., 2020 [82] Wu et al., 2020 [89] Alasham et al., 2019 [58]	Enhances problem-solving abilities Improves cognitive flexibility
Memory	Porras-García et al., 2024 [90] Yen et al., 2021 [91] Riva et al., 2020 [82] Liao et al., 2019 [92] Moreno et al., 2019 [79]	Potentially improves overall cognitive functioning
Spatial Cognition	Riva et al., 2020 [82] Maggio et al., 2019 [93] Montana et al., 2019 [94]	Enhances spatial navigation and orientation
Body Perception	Gur et al., 2024 [95] Harvie et al., 2024 [96] Morales-Tejeda et al., 2020 [97] Matamala-Gomez et al., 2019 [98]	Improves distorted body schema Reduces pain-related fear of movement
Emotional Regulation	Goudmann et al., 2022 [70] García et al., 2022 [99] Sharifpour et al., 2021 [100] Darnall et al., 2020 [101]	Improves management of pain-related negative affect Enhances overall emotional coping skills such as pain self-efficacy
Pain-Related Worries	Gava et al., 2022 [102] Ceko et al., 2022 [84]	Reduces negative thinking about pain

## Data Availability

No new data were created in this research.

## Abbreviations

The following abbreviations are used in this manuscript:

XR	Extended Reality
ADL	Activities of Daily Living
VR	Virtual Reality
MR	Mixed Reality
AR	Augmented Reality
ICD-11	International Classification of Diseases
IASP	International Association for the Study of Pain
OUDs	Opioid-Related Use Disorders
